# University College Hospital

**Published:** 1902-05-17

**Authors:** 


### UNIVERSITY COLLEGE HOSPITAL.

### THE WORK OF THE YEAR.

THE new buildings are making satisfactory progress; the pathological department on the scientific floor was opened in January, 1901, and is in full working order; the remaining two floors of the nurses' wing, the residents' floor, consisting of bed and sitting rooms and general sitting-room, and new kitchens for the hospital and nursing departments have also been opened. It is hoped that the south-eastern block, comprising five floors of wards, part of the dispensary, steward's stores, nurses' and servants' dining-rooms, students' rooms, and obstetric out-patients' departments, etc, will be completed this year. The committee hope that the remaining portion of the old hospital will be pulled down towards the end of the year to make room for the last ward block, the main entrance, and part of the out-patient department and dispensary. The sum of £15,000 is required before the Private Nursing Home can be established on the ground in University Street. The new buildings, plans of which appeared in THE HOSPITAL for April 20th, 1901, are being carried out through the liberality of Sir J. Blundell Maple, M.P.

There has been a slight increase under the head of subscriptions, which were £2,346 in 1900 and £2,398 in 1901, but donations have decreased by £272. The Hospital Sunday Fund gave £1,133; the Saturday Fund gave £283 15s. from their 1900 collection; King Edward's Fund awarded £1,400; the People's Contribution Fund sent a donation of £450. Legacies amounted to £16,183, £2,500 being for investment. The total ordinary income was £13,935, extraordinary £16,611. A bed and a cot have been endowed, the one by a former student, the other by a member of the Ladies' Association. No festival dinner was held last year, but the special appeal, a copy of which appeared in these columns, resulted in £30 in new subscriptions, and £1,255 in donations. The Christmas appeal brought in £91 in new subscriptions and £855 in donations. The total cost of up-keep, paid or owing, was £25,009. This was an increase of nearly £5,000 over 1900, and the cost of maintenance has gone up, on the whole, very considerably. This sum has only once been exceeded—in 1884, including building and leasehold expenses. High prices partly account for it, but the chief item is fuel and the cost of working the new building in conjunction with the old. It is not fair to quote the price per bed, as the average is taken over the whole building, and although when the 300 beds are opened the total cost will be increased, the cost per bed will be materially diminished. It is estimated that over £30,000 per annum will then be required to carry on the work of the hospital. The reliable income is £8,000. 2,546 patients were admitted.

